# RAP80 is an independent prognosis biomarker for the outcome of patients with esophageal squamous cell carcinoma

**DOI:** 10.1038/s41419-017-0177-2

**Published:** 2018-02-02

**Authors:** Qingyuan Yang, Wanrun Lin, Zhiwei Liu, Jiabei Zhu, Nan Huang, Zhongqi Cui, Zeping Han, Qiuhui Pan, Ajay Goel, Fenyong Sun

**Affiliations:** 1 Department of Clinical Laboratory Medicine, Shanghai Tenth People’s Hospital of Tongji University, Shanghai, China; 2 Center for Gastrointestinal Research, Center for Epigenetics, Cancer Prevention and Cancer Genomics, Baylor Research Institute and Sammons Cancer Center, Baylor University Medical Center, Dallas, TX USA; 3 Department of Laboratory, Central Hospital of Panyu, Guangzhou, Guangdong China; 4 Department of Laboratory Medicine, Shanghai Children’s Medical Center, Shanghai Jiaotong University School of Medicine, Shanghai, China

## Abstract

Esophageal squamous cell carcinoma (ESCC) is the most popular pathology of esophageal cancer (EC) in China, especially in Henan province, mid-east of China. Presently, targeting DNA damage repair (DDR) factors is a promising approach for cancer therapy. Our group has been focusing on exploring the DDR factors overexpressed in ESCC tissues to provide potential targets for therapies for many years. RAP80/UIMC1 (ubiquitin interaction motif containing 1), one of those DDR factors we tested, was highly overexpressed in ESCC tissues compared with adjacent normal tissues. Moreover, the RAP80 mRNA level was validated to be an independent prognosis biomarker for the overall survival time of ESCC patients. The following biological assays revealed that it promoted cell proliferation both in vitro and in vivo, inhibited cell apoptosis at both early and late stages, and participated in G2/M checkpoint regulation. Even though studies have reported that ATM phosphorylates RAP80 at different serine sites upon DNA damage, the reversal regulation of RAP80 on the activity of ATM has never been investigated. In the study, mechanism explorations revealed that RAP80 positively regulated the ATM activity via proteasome–ubiquitination pathway to promote the transition of G2/M phase in cell cycle. By examining a number of E3 ubiquitination ligases (Ub) and deubiquitination (DUb) enzymes, we found that RAP80 positively regulated the stability of USP13 to promote cell proliferation of EC cells. Moreover, inhibition of RAP80 greatly sensitized EC cells to ATM inhibitor KU-55933, triggering a potential combination of RAP80 inhibitors and ATM inhibitors to enhance the therapeutic efficiency of ESCC patients for the clinicians.

## Introduction

The mortality of patients with esophageal squamous cell carcinoma (ESCC), which accounts for more than 95% of esophageal cancer (EC) in China, is the highest in northeast regions of China^1^. Due to the deficiency of efficient biomarkers for early diagnosis and effective drugs, the 5-year survival rate of EC patients is <10%^2^. Therefore, it is of great importance to elucidate the accurate pathogenesis, find out novel molecular biomarkers, and provide new drug targets for ESCC patients, especially for Chinese. Classically, the radiation therapies or genotoxic chemotherapies have been exploited to treat patients with tumors lacking DDR functions to offer a greater therapeutic window^3^. Therefore, identification of DDR factors upregulated in ESCC tissues is a promising way to discover potential biomarkers and/or targets to help clinicians screen, diagnose, and develop new drugs at an early stage.

By screening a panel of DDR factors using the immunohistochemistry assays (IHC) in 100 paired ESCC tissues and adjacent normal tissues, we found that the expression of RAP80/UIMC1 was highly elevated in ESCC tissues. The Pearson *χ*^2^ analysis showed that overexpression of RAP80 in ESCC tissues was closely related with age, gender, position of tumors, gross pathology, tumor size, infiltration depth, and cell differentiation. Wang et al. report that RAP80-CCDC98/ABRAXAS forms a novel complex with BRCA1-BARD1 to participate in regulating DNA damage resistance and G2/M checkpoint, which firing its studies in DDR^4^. Our in vitro and in vivo results displayed that it significantly promoted cell growth, participated in G2/M checkpoint regulation and inhibited cell apoptosis in EC cells.

It is well known that ATM is a serine/threonine kinase activated by DNA damage and then phosphorylates multiple factors, including BRCA1, MDC1, H2AX, P53, RAP80, ATR, and CHK1/2^5,6^. Functional explorations have revealed that ATM also regulates DDR-associated checkpoint proteins, including BRCA1^7^, CHK2^8^, deoxycytidine kinase^9^, and RAD17^10^ to play a pivotal role in G2/M checkpoint. Although RAP80 is a substrate of ATM and is also involved in G2/M checkpoint control in response to DNA damage, whether RAP80 plays a reversed regulation on ATM activity in unstressed cancer cells has not been explored. In the study, we found that it positively regulated the stability of activated ATM, illustrated by the expression of phosphorylated ATM at serine 1981 site (pATM), via the ubiquitin–proteasome pathway in EC cells. Particularly, colony formation assays showed that EC cells deficient of RAP80 were sensitive to ATM inhibitor KU-55933. And further studies revealed that it also promoted cell growth of EC cells by stabilizing DUB enzyme USP13. In summary, our findings support a possible combination of RAP80 inhibitors with ATM inhibitors or USP13 inhibitors to enhance the therapeutic efficiency for ESCC patients.

## Results

### RAP80 is highly overexpressed and closely correlated with clinical properties in patients with esophageal squamous cell carcinoma

**Fig. 1:**
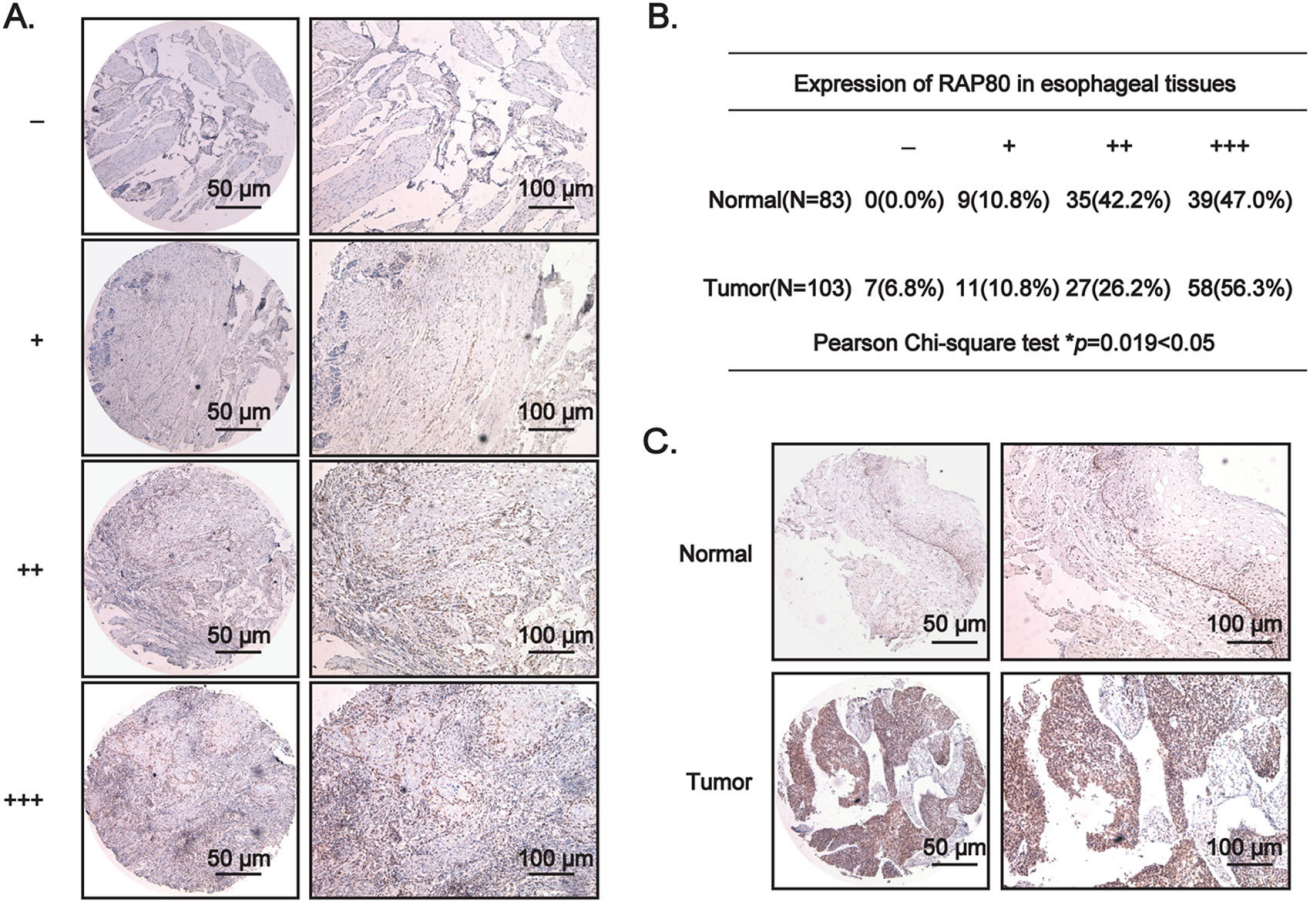
RAP80 is highly overexpressed in esophageal squamous tumor tissues. **a** The criteria for defining the expression level of RAP80 in the tissue microarray (TMA), detected by IHC staining, based on the percentage of cells with different staining intensities at two magnifications (50 and 100 μm). **b** The Pearson *χ*^2^ test of RAP80 in esophageal normal tissues (*n* = 83) and tumor tissues (*n* = 103). *p = 0.019. **c** The representative pictures of RAP80 staining in TMA. Scale bar, 50 and 100 μm, respectively

ESCC is popular with people in China, especially in Henan, Zhengzhou province. Targeting DDR factors to increase the chemo- and/or radio-sensitivity of cancer cells is an effective approach in cancer treatments^3^. Therefore, to discover potential targets or biomarkers for personalized therapy or diagnosis, our group has focused on exploring oncogenic or overexpressed DDR factors, especially the homologous recombination repair factors, in ESCC using the tissue microarrays (TMAs) for a long period. According to the grading criteria (Fig. 1a) and the statistical analysis, the percentage of ESCC tissues stained with the strongest intensity (+++) were obviously more than that of normal tissues, suggesting that RAP80 was highly elevated in ESCC tissues (Fig. 1b). The representative results were shown in Fig. 1c.

Afterwards, we further analyzed the correlation between the clinical properties and the expression of RAP80 in both normal and tumor tissues. As shown in Table 1, the overexpressed RAP80 in ESCC tissues was closely related with age, gender, occurrence position of tumor, gross pathology, tumor size, infiltration depth, and cell differentiation, implicating that RAP80 could be used as a promising predictor for the development of ESCC.

**Table 1:** The statistical analysis of relationship between clinical features and the expression of RAP80 in esophageal tissues

	RAP80 expression in esophageal tissues											
	Normal tissues					Tumor tissues						
	Total	−	+	++	+++	Total	−	+	++	+++	*χ2 test value*	*p value*
Age(years)												
<60	21	0(0%)	1(4.8%)	12(57.1%)	8(38.1%)	23	4(17.4%)	2(8.7%)	4(17.4%)	13(56.5%)	9.452	**0.024**
≥60	47	0(0%)	7(14.9%)	18(38.3%)	22(46.8%)	47	2(4.3%)	7(14.9%)	14(29.8%)	24(51.1%)	2.587	0.46
Gender												
Male	42	0(0%)	5(11.9%)	18(42.9%)	19(45.2%)	44	5(11.4%)	8(18.2%)	8(18.2%)	23(52.3%)	9.878	**0.02**
Female	26	0(0%)	3(11.5%)	12(46.2%)	11(42.3%)	26	1(3.8%)	1(3.8%)	10(38.5%)	14(53.8%)	2.542	0.468
Position of tumor												
Upper	11	0(0%)	1(9.1%)	4(36.4%)	6(54.5%)	11	1(9.1%)	4(36.4%)	4(36.4%)	2(18.2%)	4.8	0.187
Middle	48	0(0%)	5(10.4%)	22(45.8%)	21(43.8%)	48	4(8.3%)	3(6.3%)	10(20.8%)	31(64.6%)	10.923	**0.012**
Lower	9	0(0%)	2(22.2%)	4(44.4%)	3(33.3%)	11	1(9.1%)	2(18.2%)	4(36.4%)	4(36.4%)	0.952	0.813
Gross pathology												
Medullary	26	0(0%)	5(19.2%)	12(46.2%)	9(34.6%)	27	3(11.1%)	2(7.4%)	6(22.2%)	16(59.3%)	8.23	**0.041**
Fungating	3	0(0%)	2(66.7%)	0(0%)	1(33.3%)	3	0(0%)	0(0%)	1(33.3%)	2(66.7%)	3.333	0.189
Ulcerative	34	0(0%)	1(2.9%)	17(50%)	16(47.1%)	34	2(5.9%)	6(17.6%)	8(23.5%)	18(52.9%)	8.929	**0.03**
TNM stage												
I	2	0(0%)	0(0%)	0(0%)	2(100%)	2	0(0%)	0(0%)	0(0%)	2(100%)	–	–
II	30	0(0%)	4(13.3%)	12(40%)	14(46.7%)	30	3(10%)	3(10%)	9(30%)	15(50%)	3.606	0.307
III	35	0(0%)	4(11.4%)	17(48.6%)	14(40%)	35	2(5.7%)	5(14.3%)	9(25.7%)	19(54.3%)	5.33	0.149
Tumor size (cm^3^)												
<10	23	0(0%)	2(8.7%)	10(43.5%)	11(47.8%)	23	1(4.3%)	3(13%)	8(34.8%)	11(47.8%)	1.422	0.7
≥10, ≤20	29	0(0%)	6(20.7%)	14(48.3%)	9(31%)	30	1(3.3%)	3(10%)	7(23.3%)	19(63.3%)	7.89	**0.048**
>20	16	0(0%)	0(0%)	6(37.5%)	10(62.5%)	17	4(23.5%)	3(17.6%)	3(17.6%)	7(41.2%)	8.507	**0.037**
Infiltration depth												
Submucosa	3	0(0%)	0(0%)	2(66.7%)	1(33.3%)	3	1(33.3%)	1(33.3%)	1(33.3%)	0(0%)	3.333	0.343
Muscularis	12	0(0%)	2(16.7%)	3(25%)	7(58.3%)	12	1(8.3%)	1(8.3%)	5(41.7%)	5(41.7%)	2.167	0.539
Fibrosa	53	0(0%)	6(11.3%)	25(47.2%)	22(41.5%)	54	4(7.4%)	6(11.1%)	12(22.2%)	32(59.3%)	10.411	**0.015**
Lymphatic metastasis												
Negative	41	0(0%)	7(17.1%)	17(41.5%)	17(41.5%)	41	3(7.3%)	5(12.2%)	10(24.4%)	23(56.1%)	6.048	0.109
Positive	27	0(0%)	1(3.7%)	13(48.1%)	13(48.1%)	29	3(10.3%)	4(13.8%)	8(27.6%)	14(48.3%)	5.964	0.113
Cell differentiation												
Undifferentiated	4	0(0%)	0(0%)	2(50%)	2(50%)	4	1(25%)	0(0%)	1(25%)	2(50%)	1.333	0.513
Low	12	0(0%)	2(16.7%)	4(33.3%)	6(50%)	12	1(8.3%)	2(16.6%)	4(33.3%)	5(41.7%)	1.091	0.779
Middle	22	0(0%)	1(4.5%)	13(59.1%)	8(36.4%)	24	3(12.5%)	3(12.5%)	5(20.8%)	13(54.2%)	8.675	**0.034**
High	30	0(0%)	5(16.7%)	11(36.7%)	14(46.7%)	30	1(3.3%)	4(13.3%)	8(26.7%)	17(56.7%)	1.875	0.599

The bold values indicate *p* values  < 0.05 of the Chi-square test.

### RAP80 promotes cell growth, inhibits cell apoptosis, and participates in G2/M checkpoint control in esophageal cancer cells

**Fig. 2:**
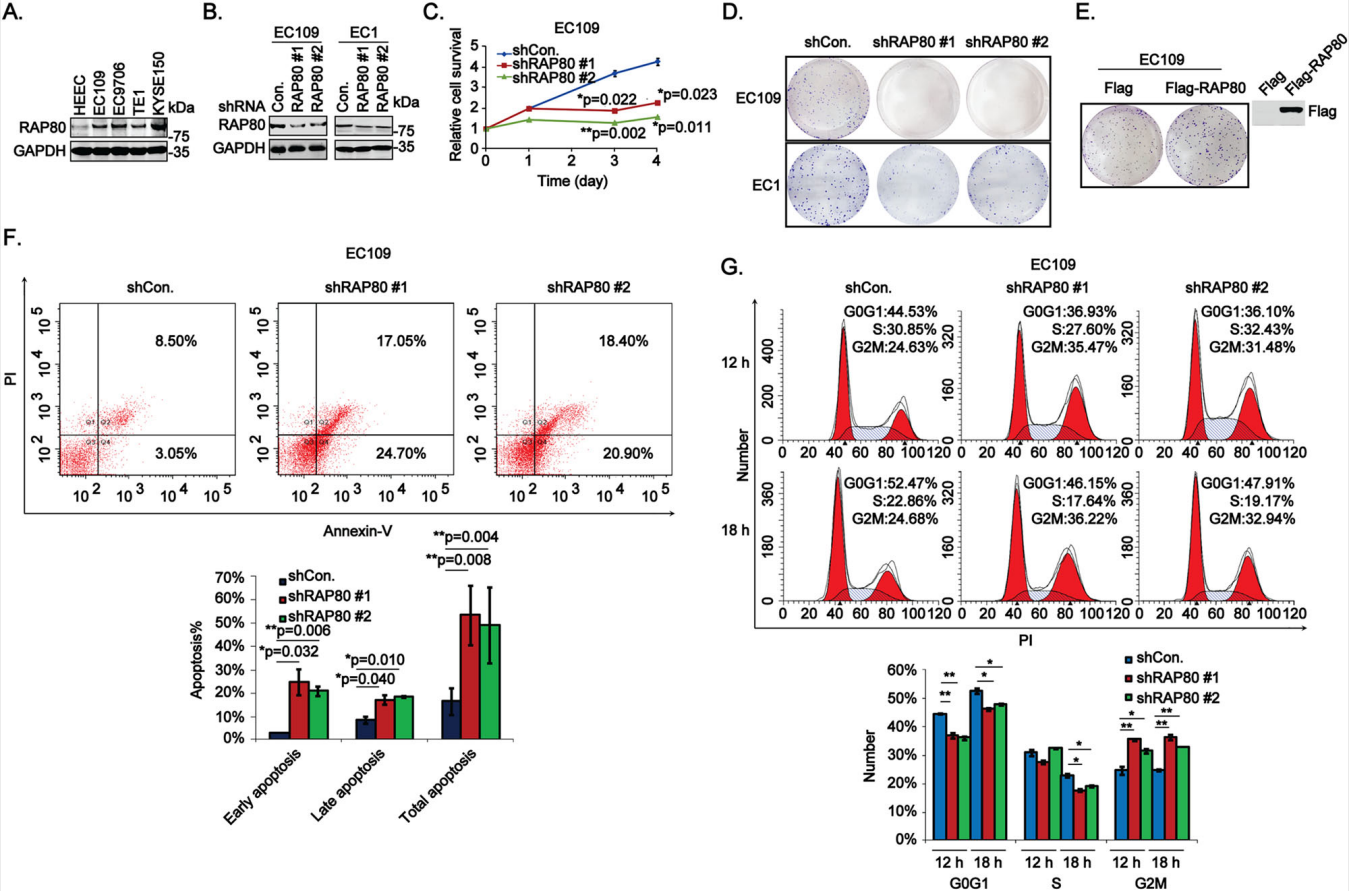
Inhibition of RAP80 greatly attenuates cell proliferation, arrests cells at G2/M phase, and promotes cell apoptosis in vitro. **a** The whole-protein extracted from EC cells, including EC109, EC9706, TE1, and KYSE150, and an immortalized epithelial esophageal cell line HEEC were subjected to western blotting assays to explore the expression of RAP80 in these cells, taking GAPDH as the internal calibrator. **b** The knockdown efficiency of RAP80 using shRNAs in EC109 and EC1 cells were verified using the western blotting assays. **c** The RAP80 stably depleted EC109 cells (EC109/shRAP80 #1), taking EC109/shCon. as a negative control, were subjected to MTT analysis to evaluate the role of RAP80 in cell proliferation. **d** Cell pellets of stable RAP80 knockdown cell lines EC109 and EC1 were subjected to colony formation assays to evaluate the role of RAP80 in cell growth. **e** EC109 cells transfected with Flag or Flag-RAP80 were subjected to colony formation assays. The transfection efficiency was confirmed by western blotting assays with specific antibody to Flag. **f** Cell apoptosis analysis of EC109/shCon. and EC109/shRAP80 #1, #2 cells using flow cytometry assays. Q2 late apoptosis, Q4 early apoptosis, Q2 + Q4 total apoptosis. **g** Cell cycle analysis of EC109/shCon. and EC109/shRAP80 #1, #2 cells starved in FBS-free medium for 12 h, followed by the recovery in fresh medium for 12 h and 18 h, respectively. *p < 0.05, **p < 0.01

Similar to above tissue results, RAP80 was obviously overexpressed in EC cells as well (Fig. 2a). Next, the EC cells stably transfected with shCon. or shRAP80 #1, #2, the interfering efficiency of which was confirmed in Fig. 2b, were used to study the biological roles of RAP80. Results from cell survival analysis revealed that downregulation of RAP80 greatly inhibited the proliferation of EC109 cells (Fig. 2c). Besides, the colony formation results showed that the growth of EC cells were remarkably reduced in RAP80-depleted cells (Fig. 2d) but greatly increased in it overexpressed cells (Fig. 2e), supporting a positive regulation of it in EC progression. Moreover, data from flow cytometry assays showed that RAP80-negative-regulated cell apoptosis at both early and late stage (Fig. 2f). Alternatively, similar to other HRR factors^11^, RAP80 was also involved in regulating G2/M checkpoint (Fig. 2g).

**Fig. 3:**
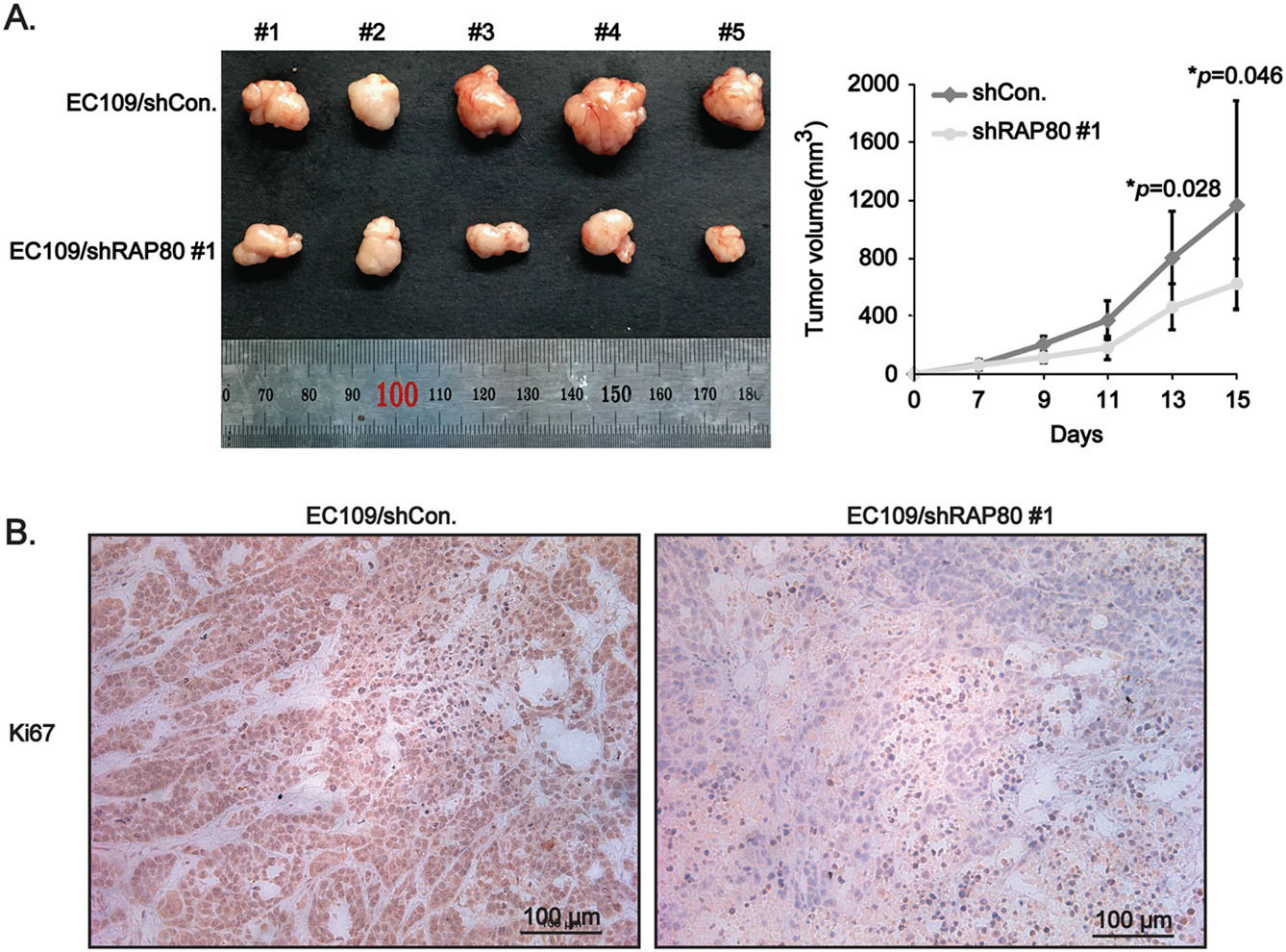
RAP80 significantly promotes growth of EC cells in vivo. **a** Tumors dissected from nude mice injected with EC109/shCon. and EC109/shRAP80 #1 cells on the different body sides (*n* = 5). The tumor volumes (mm^3^) on the indicated days were graphically depicted as tumor growth curve. **b** Representative IHC staining of proliferative marker Ki67 in the tumor tissues dissected from nude mice. Scale bar, 100 μm

Afterwards, we performed the xenograft tumor-bearing experiments to observe the growth of EC cells in vivo. As shown in Fig. 3a, the tumor volumes of EC109/shRAP80 #1 cells were significantly smaller than those of EC109/shCon. cells. Besides, the staining of Ki67, a biomarker for cell proliferation, in the tissues from above nude mice was obviously reduced in EC109/shRAP80 #1 cells (Fig. 3b). Collectively, data from both in vitro and in vivo experiments supported an oncogenic role of RAP80 in EC tumorigenesis, which was in line with its overexpression in both ESCC tissues and EC cells.

### RAP80 deficiency inhibits the activation of ATM to enhance the sensitivity of esophageal cancer cells to ATM inhibitor KU-55933 by arresting cells at G2/M phase

**Fig. 4:**
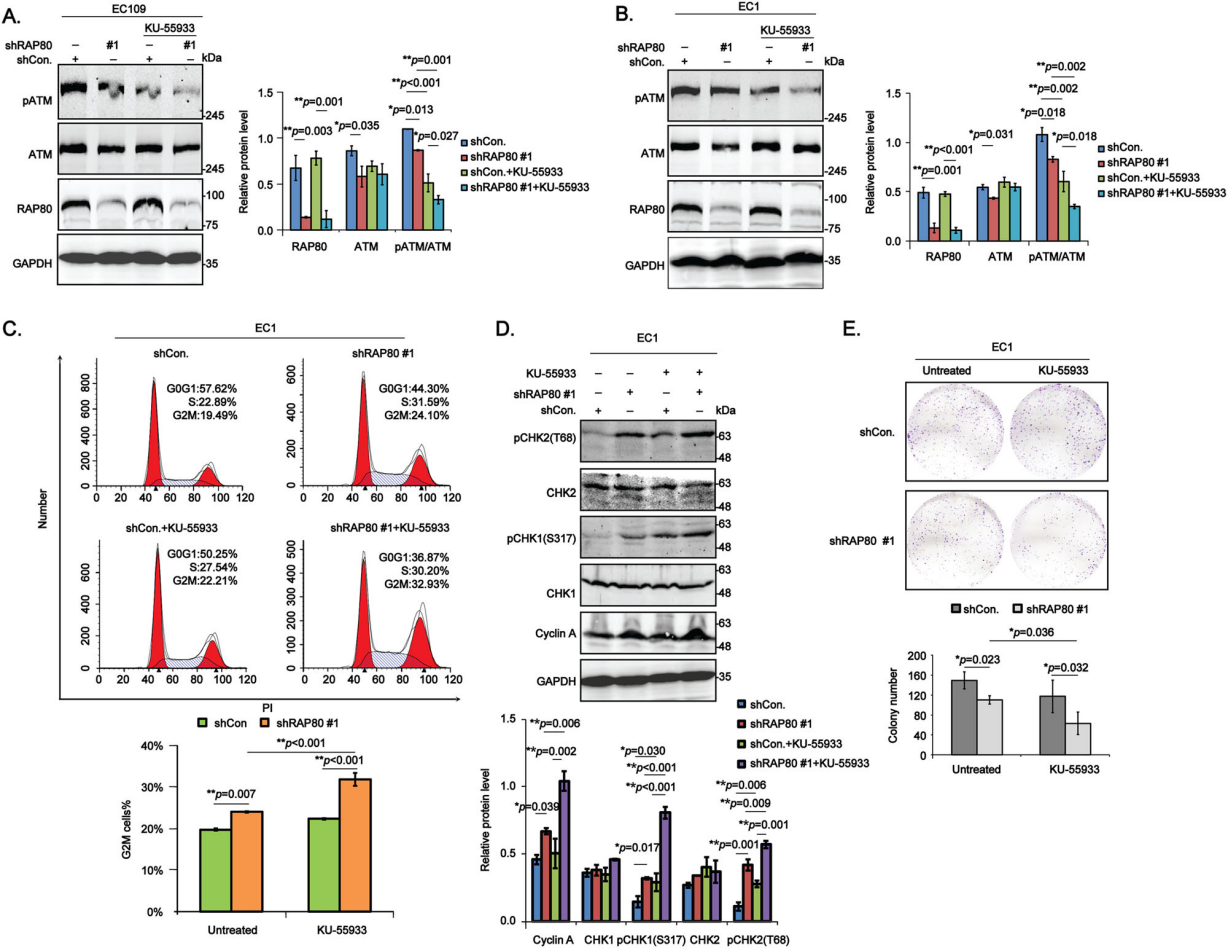
RAP80 positively regulates ATM activity to promote cell survival by facilitating the G2/M checkpoint transition. **a**, **b** The stable shRAP80 #1 infected EC cells, EC109 (**a**) and EC1 (**b**), were treated with or without ATM inhibitor KU-55933 and then lysed to detect the indicated proteins using western blotting assays. The gray values were obtained from three independent experiments using Image J software and then subjected to ANOVA test. **c** EC1/shCon. or EC1/shRAP80 #1 cells pretreated with or without KU-55933 were starved in serum-free medium for 12 h and then recovered in fresh medium for 18 h, afterwards, these pellets were collected to analyze the cell cycle distribution using flow cytometry. The percentage of cells in G2/M phase from three independent experiments was represented as mean ± STD and statistically analyzed using the ANOVA test. **d** Pellets from above FACS analysis were lysed and subjected to examine G2/M checkpoint-specific proteins using western blotting assays. E. EC1/shCon. and EC1/shRAP80 #1 cells treated with or without ATM inhibitor KU-55933 were subjected to colony formation assays

In response to DNA damage, RAP80 is phosphorylated by ATM/ATR at numerous serine sites (S101, S205, S140, S402, S419)^4,12^ and then forms a pivotal complex with CCDC98-BRCC45-MERIT40-BRCC36-NBA1-BRCA1 (BRCA-A) to regulate the G2/M checkpoint and the cellular sensitivity to irradiation (IR)^13,14^. In the study, we explored the effects of RAP80 on the activity of ATM, indicated by the ratio of pATM to ATM (pATM/ATM), in unstressed EC cells, which has never been investigated by now. Intriguingly, the expression of pATM/ATM was obviously reduced in shRAP80 #1 infected EC cells, especially when treated with KU-55933 (Fig. 4a, b). Moreover, overexpression of RAP80 in EC cells greatly increased the activity of ATM (Figure S1A), confirming its positive regulation on ATM activity. To exclude the possibility that RAP80-regulated ATM activity at the transcriptional level, we detected the mRNA level of ATM in EC cells transfected with shCon. or shRAP80 #1 using qPCR method, the results of which showed no difference in both EC1 and EC109 cells (Figure S1B).

According to previous studies, ATM activity is required for G2/M checkpoint control^15^. Our above data showed that RAP80 positively regulated G2/M checkpoint transition and ATM activity, thus it is reasonable to think that it is likely to regulate the G2/M phase transition by activating ATM. Results from flow cytometry showed that EC1/shRAP80 #1 cells were significantly arrested in G2/M phase, especially when treated with KU-55933 (Fig. 4c). Besides, the following western blotting assays of G2/M checkpoint-specific proteins, such as CHK1/2, Cyclin A, further confirmed the G2/M cell cycle arrest in EC1/shRAP80 #1 cells, particularly in response to KU-55933 treatment (Fig. 4d). Above all, we concluded that RAP80 positively regulated G2/M checkpoint transition by activating ATM in EC cells.

The small and selective inhibitors of ATM have been suggested to be used individually and/or in combination with regular tumor therapeutic agents such as chemo-sensitizers and radio-sensitizers^16^. In the study, data from colonial survival assays showed that ATM inhibitor KU-55933 strongly enhanced the cell growth inhibition of EC1/shRAP80 #1 cells(Fig. 4e), uncovering a possible combination therapy of RAP80 inhibitors and ATM inhibitors for ESCC patients.

### RAP80 stabilizes USP13 in the ubiquitin–proteasome way to promote growth of esophageal cancer cells

**Fig. 5:**
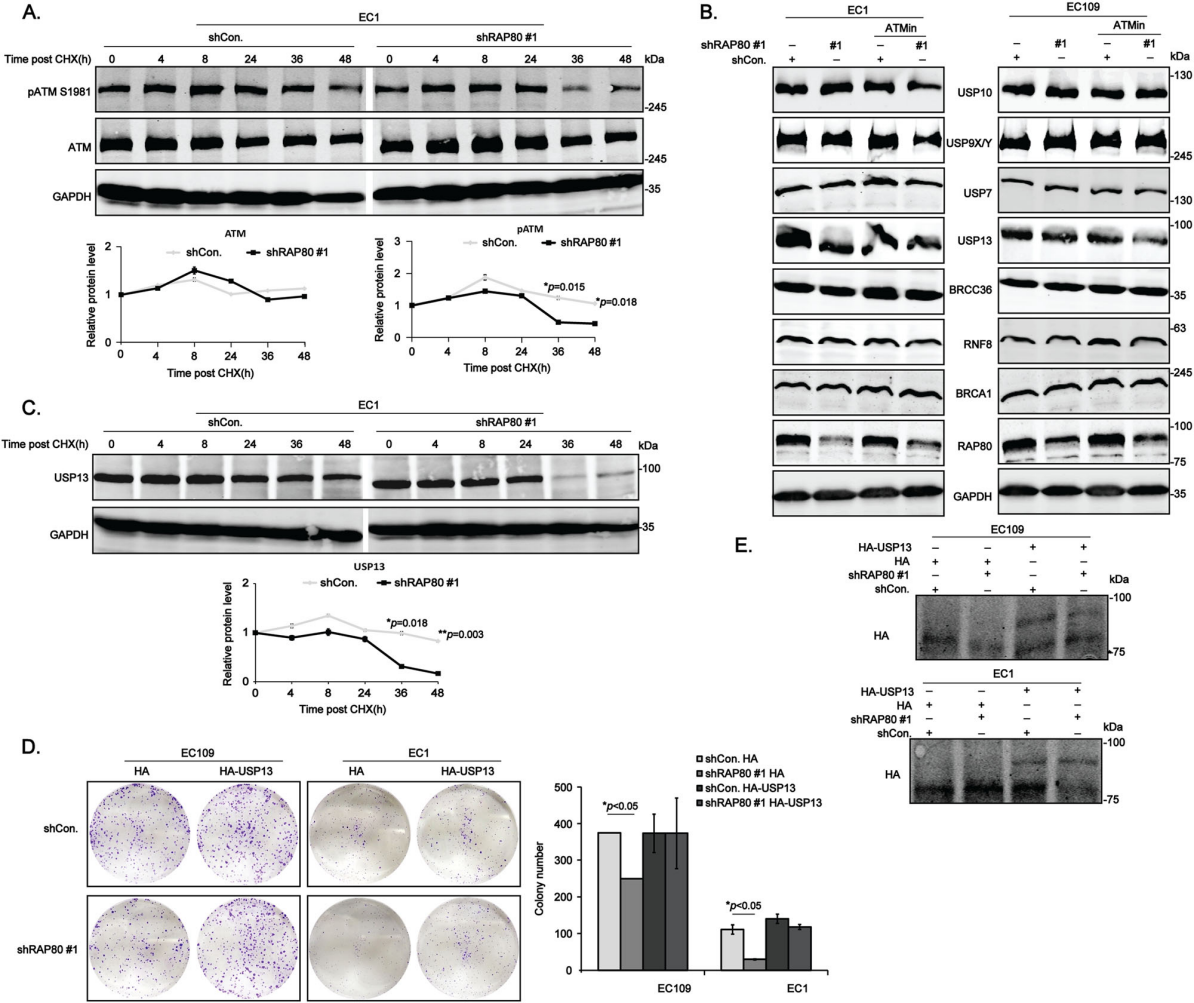
RAP80 stabilizes USP13 in a ubiquitin–proteasome pathway to promote growth of EC cells. **a** The whole-protein extracted from EC1/shCon. and EC1/shRAP80 #1 cells treated with 0.2 mg/ml CHX for indicated time points were subjected to western blot analysis to test the indicated proteins. The gray values were statistically analyzed. **b** The screening tests of E3 ligases and deubiquitylated enzymes in shCon. and shRAP80 #1 infected EC109 and EC1 cells using western blotting assays. **c** The whole-protein extracted from EC1/shCon. and EC1/shRAP80 #1 cells treated with 0.2 mg/ml CHX for indicated time points were subjected to western blot analysis to evaluate the stability of USP13. **d**, **e** The stable RAP80 knockdown EC109 and EC1 cells transfected with HA or HA-USP13 were subjected to colony formation analysis (**d**), the transfection efficiency was confirmed using western blotting assays (**e**)

The critical function of RAP80 is to target a complex including the E3 ligases BRCA1-BARD1 and the deubiquitinating enzyme (DUB) BRCC36 to lysine 63-linked ubiquitin polymers at DNA damage sites^12^, suggesting that it plays a prominent role in the ubiquitin–proteasome signaling. Thus, we considered that RAP80 regulated the activity of ATM in the ubiquitin–proteasome manner. Intriguingly, the expression of pATM was significantly reduced in EC1/shRAP80 #1 cells at 36 h post the treatment of cycloheximide (CHX), an inhibitor of protein synthesis, whereas it started to reduce at 48 h in EC1/shCon. cells (Fig. 5a), suggesting that RAP80 positively regulated the stability of pATM in unstressed EC cells in the ubiquitin–proteasome pathway.

The fact that RAP80 is a ubiquitin-interacted protein, but not an E3 ligase or DUB enzyme, it is impossible to directly regulate the stability of pATM. As a result, we screened a number of E3 ligases and DUB enzymes using western blotting assays to discover the direst ubiquitin-regulating proteins connecting RAP80 and pATM. Interestingly, similar to pATM, the expression of USP13 was also reduced in EC cells infected with shRAP80 #1, which could be further enhanced by KU-55933 (Fig. 5b), suggesting that RAP80 might regulate the expression of USP13 to manipulate the stability of pATM. Unfortunately, the rescue analysis showed that re-expression of HA-USP13 was not sufficient to reverse the reduction of pATM in RAP80-depleted EC cells (Figure S1C and D), leading us to think that there are some other E3 ligases or DUB enzymes involved in mediating the regulation of RAP80 on the degradation of pATM.

Afterwards, the stability of USP13 in EC1/shCon. and EC1/shRAP80 #1 was further investigated. Surprisingly, like pATM, the expression of USP13 was dramatically reduced in EC1/shRAP80 #1 cells at 36 h post CHX treatment (Fig. 5c). Additionally, MG-132, a kind of proteasome inhibitor, sufficiently rescued the reduction of both pATM and USP13, not the ATM, in EC1/shRAP80 #1 cells (Figure S1E), suggesting that RAP80 manipulated the stability of both pATM and USP13 in a ubiquitin–proteasome manner at the same time. Recently, some scientists have reported that ATM is activated to phosphorylate USP13 at Thr196 site in response to DNA damage, stabilizing RAP80 to assemble BRCA1 to repair DSBs^17^. Therefore, our data could be explained that RAP80 presented a positive feedback on the activity of ATM as well as the expression of USP13, ensuring a safer environment for the growth of EC cells by efficiently repairing endogenous DSBs. Importantly, the following colony formation assays demonstrated that RAP80 positively regulated the growth of EC cells via USP13 (Fig. 5d), the overexpression efficiency of which was confirmed by western blotting assays (Fig. 5e).

### Downregulation of RAP80 prominently generates more DNA damage and impairs the recruitment of BRCA1 in response to cisplatin treatment

**Fig. 6:**
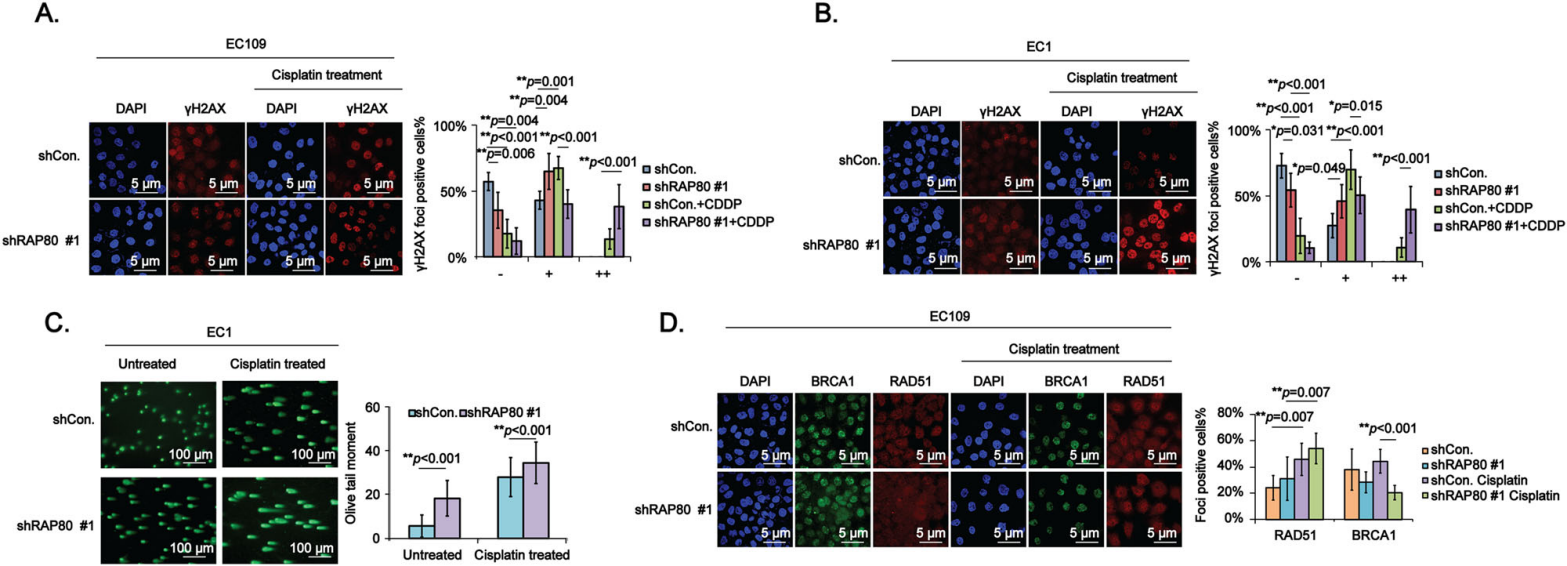
RAP80 deficiency remarkably sensitizes EC cells to cisplatin treatment. **a**, **b** EC109 (**a**) and EC1 (**b**) cells infected with shCon. or shRAP80 #1 were treated with or without cisplatin and then subjected to immunofluorescence (IF) assays to test the formation of γH2AX foci. The percentage of γH2AX foci-positive cells at different levels (−,+, ++) was statistically analyzed. Representative results were shown. Scale bar, 5 μm. **c** EC1/shCon. and EC1/shRAP80 #1 cells treated with or without cisplatin were subjected to the neutral comet assays. The olive tail moment was used to assess the damage severity. **d** EC109/shCon. and EC109/shRAP80 #1 cells treated with or without cisplatin were subjected to IF assays to test the BRCA1 (Green) and RAD51 (Red) foci formation. Representative results were shown. Scale bar, 5 μm

It is reported that the foci formation of γH2AX is positively correlated with the severity of DNA damage both in vivo and in vitro^18^. Results from our confocal IF assays showed that γH2AX foci-positive cells in 200 cells were significantly increased in shRAP80 #1 transfected cells, especially when treated with cisplatin (Fig. 6a, b). Besides, the neutral comet assay results further consolidated the above data (Fig. 6c). Taken together, RAP80 helped EC cells survive from intrinsic and extrinsic damage by repairing the damaged DNA.

In response to DNA damage, the DDR factor RNF8 interacts with E2 enzyme UBC13 to ubiquitinate H2AX, specifically the K63 sites, the structure of which is critical for RAP80 to recruit the canonical BRCA-A complex which then assemble the downstream recombinase RAD51 to repair the damage sites^11^, suggesting that RAP80 is a critical for the assembly of BRCA1 and RAD51. Unexpectedly, results from our confocal IF assays showed that the recruitment of BRCA1 was strikingly impaired in EC109/shRAP80 #1 cells treated with cisplatin (Fig. 6d, green), whereas RAD51 was still efficiently recruited (Fig. 6d, red). This phenomenon might be attributed to the fact that there are a lot of other factors synergistically recruiting RAD51 to the damage sites^19–21^.

Above all, our studies showed that the oncogenic protein RAP80 promoted esophageal tumorigenesis in two ways, one is that it governs the stability of pATM to facilitate the G2/M checkpoint transition and helps EC cells survive from ATM inhibitor KU-55933, the other is that it stabilizes USP13 to accelerate the growth of EC cells. However, the axis RAP80-ATM-USP13 in manipulating the development of esophageal cancer still requires further investigation in the future.

### RAP80 independently predicts a poor outcome for patients with esophageal squamous cell carcinoma

Afterwards, we further enrolled patients with ESCC from Japan, including a training cohort and a validation cohort, to evaluate the potential of RAP80 as a predictor for the outcome of ESCC patients. The correlation between RAP80 mRNA level and clinical information were shown in Table S1 and Table S2, respectively. Unlike RAP80 protein, there was no significant correlation between RAP80 mRNA level and the clinicopathological parameters in the two cohorts.

**Fig. 7:**
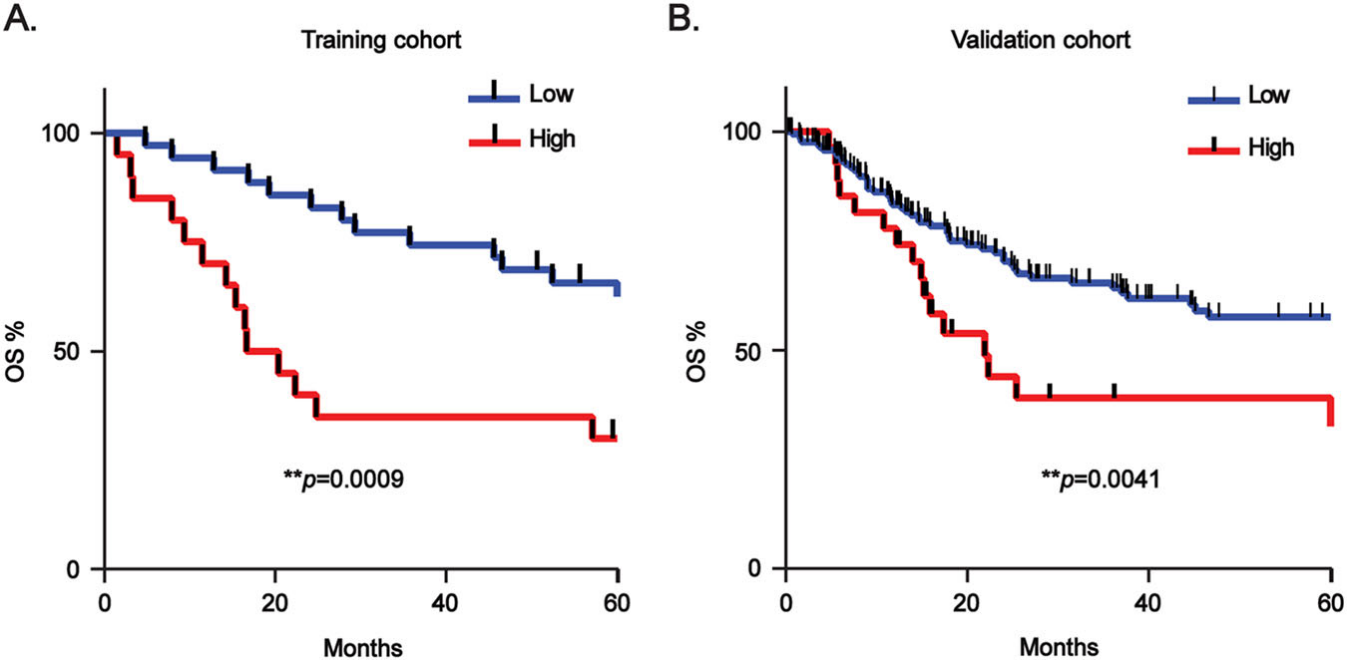
Overexpression of RAP80 mRNA independently predicts a poor outcome of ESCC patients. **a**, **b** Kaplan–Meier survival curves stratified by RAP80 mRNA level in training cohort (**a**) and validation cohort (**b**)

Alternatively, the correlation of RAP80 mRNA level with OS was separately analyzed in the two cohorts. The cutoff for optimal *p* value was calculated by x-tile, which was used to categorize RAP80 expression levels in tumor tissues as low or high. OS curves were plotted according to RAP80 mRNA levels using the Kaplan–Meier method. As presented in Fig. 7a, b, the patients with high RAP80 expression exhibited a significantly poorer outcome than those with low RAP80 expression (**p = 0.0009 in training cohort and **p = 0.0041 in validation cohort). For the univariate analysis of OS, in the training cohort, the relative level of RAP80 mRNA expression (**p = 0.001), tumor size (*p = 0.025), lymphatic invasion (*p = 0.024), lymph nodes metastasis (*p = 0.011), and tumor stage (**p = 0.006) were prognostic indicators (Table 2). In the validation cohort, the RAP80 mRNA expression (**p = 0.004), lymphatic invasion (**p = 0.002), lymph nodes metastasis (**p = 0.002), and tumor stage (**p < 0.001) were prognostic indicators (Table 3). For the multivariate analysis, in the training cohort, RAP80 mRNA level was an only independent prognostic indicator for the OS of ESCC patients (**p = 0.005, Table 2). In the validation cohort, in addition to the tumor stage (**p = 0.003), RAP80 mRNA level was also an independent prognostic indicator for the OS of ESCC patients (**p = 0.002, Table 3).

**Table 2:** Univariate and multivariate analysis of clinicopathological factors for overall survival of ESCC patients in the training cohort

Characteristics	Univariate analysis		Multivariate analysis	
	HR (95%CI)	*p* value^a^	HR (95%CI)	*p* value^a^
Gender (male/female)	1.239 (0.433–3.547)	0.689		
Size (cm) (<5/≥5)	2.886 (1.095–7.609)	0.025*	1.319 (0.438–3.970)	0.622
Differentiation (poor+others/well+moderate)	1.822 (0.880–3.771)	0.101		
Lymphatic invasion (present/absent)	2.283 (1.095–4.760)	0.024*	1.330 (0.589–3.003)	0.493
Nerve invasion (present/absent)	1.873 (0.892–3.930)	0.092		
Tumor grade (T1, T2/T3, T4)	2.051 (0.716–5.875)	0.172		
LNM (present/absent)	4.201 (1.271–13.882)	0.011*		
Distant metastasis (present/absent)	1.536 (0.361–6.532)	0.558		
TNM^b^ (I+II/III+IV)	3.589 (1.367–9.421)	0.006**	2.783 (0.910–8.507)	0.073
RAP80 (high/low)	3.155 (1.548–6.430)	0.001**	2.896 (1.382–6.069)	0.005**

*CI* confidence interval, *HR* hazard ratio, *LNM* lymph nodes metastasis

^a^ All statistical tests were two-sided

^b^ Tumor stage was obtained according to the TNM criteria. Significance level: *p < 0.05, **p < 0.01

**Table 3:** Univariate and multivariate analysis of clinicopathological factors for overall survival of ESCC patients in the validation cohort

Characteristics	Univariate analysis		Multivariate analysis	
	HR (95%CI)	*p* value	HR (95%CI)	*p* value
Gender (male/female)	1.717 (0.942–3.128)	0.074		
Age (≤60/>60)	1.034 (0.619–1.726)	0.900		
Size (cm) (≤5/>5)	1.247 (0.765–2.030)	0.375		
Differentiation (poor+others/well+moderate)	1.351 (0.741–2.463)	0.324		
Lymphatic invasion (present/absent)	2.843 (1.414–5.716)	0.002**	1.976 (0.946–4.128)	0.070
Nerve invasion (present/absent)	1.265 (0.910–1.757)	0.160		
Tumor grade (T1, T2/T3, T4)	1.386 (0.841–2.285)	0.198		
LNM (present/absent)	2.329 (1.338–4.053)	0.002**		
Distant metastasis (present/absent)	1.884 (0.964–3.681)	0.059		
TNM (I+II/ III+IV)	2.660 (1.590–4.449)	0.000**	2.341 (1.343–4.080)	0.003**
RAP80 (high/low)	2.142 (1.256–3.652)	0.004**	2.239 (1.358–3.995)	0.002**

## Discussion

ESCC is the most popular pathology of EC with people in China. Our group has been investigating the oncogenic DDR factors to discover potential targets for personalized therapy of ESCC for a long time. Using a series of TMAs, we stained a lot of DDR factors using IHC assays, and found that RAP80 was highly overexpressed in ESCC tissues, which was closely related with age, gender, occurrence position of tumor, gross pathology, tumor size, infiltration depth, and cell differentiation. Additionally, we analyzed the mRNA expression of RAP80 in two independent cohorts comprising 254 ESCC fresh frozen samples collected in Japan. The results suggested that the higher RAP80 mRNA level, the poorer ESCC prognosis. Besides, the Cox regression analysis determined that in both two cohorts, RAP80 mRNA level was an independent biomarker for predicting the OS of ESCC patients. Taken together, our findings proved that RAP80 was a novel prognostic biomarker for ESCC patients and might serve as a promising target for ESCC therapy.

Using the RAP80 knockout mice (RAP80^−/−^), Yin et al. have reported that the susceptibility of lymphomas in RAP80^−/−^ mice is nearly 14-fold of that in RAP80 wild type (WT) mice^22^, implicating a tumor suppressive role of RAP80. Paradoxically, in pancreatic cancer cells, it is reported that inhibition of RAP80 using siRNAs, the cell apoptosis is significantly induced, indicated by the expression of apoptotic biomarkers, including BAX, BCL-2, SURVIVIN, and Caspas-8 at both mRNA and protein levels^23^, revealing an oncogenic role of RAP80 in pancreatic tumorigenesis. We attribute this contradictory role of RAP80 in tumorigenesis to different studying models, one uses the normal cells, the other uses the tumor cells. In the study, our data from both in vitro and in vivo experiments supported that RAP80 was an oncogene in EC development.

It is reported that knockdown of RAP80 using siRNAs in Hela cells treated with IR showed a defective G2/M checkpoint control^24^. Moreover, proteins interacting with RAP80 have also been implicated to play critical roles in G2/M checkpoint manipulation, for instance, CCDC98^25^, BRCA1, CtIP^24^, and NBA1^13^. Upon DNA damage, RAP80 is reported to be phosphorylated at several serine sites, such as S101^12^, S140, S205, S402, S419^4^, S677^26^, which requires the involvement of ATM kinase, substrates of which have also been suggested to control the G2/M checkpoint, including BRCA1, CHK2, RAD17^15^. In the study, our data showed that inhibition of RAP80 caused a significant G2/M cell cycle arrest, which could be obviously enhanced by ATM inhibitor KU-55933, suggesting that the activated ATM is necessarily required for RAP80 to facilitate the G2/M checkpoint transition in EC cells.

Mechanistic studies on the regulation of ATM activity reveal that activated transcription factor-2, cooperating with Cul3 ubiquitin ligase, promotes the degradation of TIP60, leading to a significant reduction of ATM activity under both basal and IR-induced conditions^27^. To our present knowledge, although RAP80 is phosphorylated by ATM upon DNA damage, no one has reported the roles of RAP80 in regulating ATM activity under basal conditions. Data in our study demonstrated that it played a positive role in manipulating the expression of pATM in unstressed EC cells. Due to the close correlation of RAP80 with ubiquitin structure, the following mechanistic investigation revealed it regulated ATM activity in a ubiquitin–proteasome manner. Unfortunately, we did not find any interactions between ATM and RAP80 in cells treated with or without cisplatin (Figure S2), leading us to suppose that there were some unknown mediators transmitting the signals from RAP80 to ATM.

As we know that the ubiquitination status in mammalian is balanced by the E3 ligases which promote ubiquitination and the DUB enzymes which remove the ubiquitin chains from their targets. Thus, in a screening analysis using western blotting assays, we found that the expression of USP13 was remarkably reduced in RAP80-depleted EC cells, particularly when treated with KU-55933. Unexpectedly, USP13 could not reverse the inhibition of ATM activity in shRAP80-transfected cells. But the following stability assays showed that USP13 degraded at the same time point as pATM in RAP80 impaired cells, displaying a more complicated relationship among RAP80, USP13, and ATM. Recently, researchers from Mayo Clinic uncover that ATM is activated in response to IR damage, and then phosphorylates USP13 at Thr196 site to stabilize RAP80, leading to the efficient recruitment of BRCA1 to the damage sites^17^. Consistent with their reports, we also found that RAP80 binded with USP13 in EC cells treated with or without cisplatin (Figure S2). Therefore, based on their findings, our above results could be interpreted as that RAP80-regulated pATM stability via some unknown E3 ligases or DUB enzymes, thus stabilizing USP13 to promote the growth of EC cells. However, further studies are required to discover ubiquitin related factors mediating the regulation of RAP80 on the activity of ATM in unstressed or stressed cells.

Although RAP80 has been recognized as a key regulator in DDR signalings for a long time, no one has provided direct evidence supporting its roles in DDR. Results from neutral comet assays in this work filled this gap. Moreover, the foci formation of γH2AX in EC cells stably transfected with shCon. or shRAP80 and treated with or without cisplatin further confirmed its positive roles in regulating DDR. In line with Yunhui Li et al. findings which suggest that USP13 stabilizes RAP80 to facilitate the recruitment of BRCA1 in response to DNA damage^17^, we also found that downregulation of RAP80 significantly reduced the efficient recruitment of BRCA1 to the damage sites in response to cisplatin treatment in EC cells. Previous reports suggested that RAP80 is involved in DDR by leading BRCA-A complex as well as its downstream factor RAD51 to damage sites^11^. However, the recruitment of RAD51 was unimpaired in RAP80 knockdown cells treated with or without cisplatin. By referring to a large number of literatures which suggest that some other factors, such as CHK1^19^, RNF4^20^, Cyclin D1^21^, also participate in recruiting RAD51 recombinase to damage sites, thus we speculated that RAD51 was still efficiently recruited in RAP80 abolished cells due to the compensatory roles of other DDR factors in EC cells.

Overall, our data support the following conclusions: first, RAP80 is a novel and independent biomarker for predicting the development of ESCC; second, targeting RAP80 is a promising way for ESCC patients’ therapy; third, combination application of RAP80 inhibitors and ATM inhibitors or USP13 inhibitors will remarkably increase the therapeutic opportunities for ESCC patients in the future.

## Materials and methods

### Cell culture and treatments

EC cell lines, including EC109, EC1, EC9706, TE1, KYSE150, and the immortalized esophageal epithelial cell line HEEC were all cultured in Dulbecco’s modified Eagle’s medium (DMEM) with addition of 12% fetal bovine serum, 100 U/ml penicillin, and 100 U/ml streptomycin, and maintained at 37 °C incubator with 5% CO_2_. For the inhibition of ATM activity, the EC cells were treated with 10 μM KU-55933 (Selleckchem) for 4 h. For the protein stability analysis, the cells were treated with 0.2 mg/ml cycloheximide (CHX, Solarbio) for the indicated time points. For the damage induction, EC cells were treated with 10 μg/ml cisplatin for 12 h, followed by 2 h recovery in fresh medium. 40 μM MG-132 (Sigma) was used to treat cells for 6 h.

### Plasmids and transfection

The shRNAs against different sequences of RAP80 (shRAP80 #1, #2) were kindly presented by Prof. Zhenkun Lou lab at Mayo Clinic. The control shRNAs (shCon.) were purchased from Invitrogen. The HA-USP13 plasmids were given by Prof. Ping Wang lab at Tongji University. The eukaryotic RAP80 overexpressing plasmids (Flag-RAP80) were constructed by amplifying the open reading frame (ORF) of human RAP80 from cDNA and ligated into the vector pCMV-N-Flag (Flag). The establishment of stable RAP80 knockdown EC cells and the transfection of plasmids were performed according to our previous protocols^28^.

### Antibodies

In the study, the primary antibodies specific to RAP80, RNF8, HA, Ki67, RAD51, and RAP80 were all commercially obtained from Abcam. And BRCC36, USP13, USP7, USP9X/Y, USP10 were from Epitomics, BRCA1 was from Santa, ATM and pATM were from Cell Signaling Technology, γH2AX was from Merck Millipore, GAPDH were from Sungenebiotech.

### Flow cytometry

For the cell cycle analysis, the stable EC cells were synchronized in FBS-free medium for 12 h and then recovered in fresh medium for 12 and 18 h, respectively. Afterwards, these cells were fixed with 95% ethanol at 4 °C overnight. The next day, the pellets were washed with cold PBS for three times and then stained with PI/RNase buffer (BD Pharmingen^TM^) for 10 min at room temperature, followed by the analysis on the flow cytometry instrument (BD Bioscience). For the apoptosis analysis, the cells were stained with Annexin-V and PI (Invitrogen) in turn at room temperature, followed by the flow cytometry analysis according to our previous performance^29^.

### Immunohistochemistry assay and confocal immunofluorescence assay

To test the expression of RAP80 in ESCC tissues using IHC assays, we first collected about 100 pairs of tumor tissues and adjacent normal tissues from ESCC patients who were diagnosed between January 2009 and December 2011 at the Department of Pathology, Anyang Tumor Hospital, Fourth Affiliated Hospital of Henan of Science and Technology. Afterwards, these tissues were arrayed onto a blank paraffin block, which was then continuously sliced to a number of slides to generate the TMAs with the same clinical information^28^. Then the assays were performed according to our previously reported protocol^28,30^. The analyzing process was carried out by professional pathologists who were blind to the clinical information of these samples based on the percentage of cells with different staining intensities under two magnifications, ×50 and ×100. By re-checking the pathology of these tissues and excluding damaged tissues, finally, the number of normal tissues was 83 and the tumor tissues was 103.

For the detection of foci formation of γH2AX, RAD51, and BRCA1 in EC cells treated with or without cisplatin, the immunofluorescence assays (IF) were carried out and finally, the slides were analyzed under the confocal microscope (Zeiss). Notably, for the statistics of γH2AX foci in the nucleus, three levels were categorized according to the number of foci per cell, <5 (−), 5–50 (+), and >50 (++). Then the percentage of cells at different levels was calculated using the following formula: γH2AX foci-positive cells% = (number of cells with foci at different levels)/(the total number of cells). All the above assays were performed according to our previous reports^28^.

### In vivo proliferation assays

According to our preliminary studies^30^, about 800 × 10^4^ cells of EC109/shCon. or EC109/shRAP80 #1 were subcutaneously injected into the left side or right side of each male nude mice, aged 4 weeks (*n* = 5), commercially purchased from Shanghai Super-B&K Laboratory Animal Corp. Ltd (Shanghai, China) and cultured in SPF animal house of Shanghai Tenth People’s Hospital of Tongji University, approved by the Animal Experiment Management Committee of Shanghai. Seven days after the initial injection, the tumor volumes were started to record every two days. When the volumes reached 1000 mm^3^, these tumors were kindly dissected according to the required ethics and pictured before being fixed with 4% paraformaldehyde, embedded in paraffin fax for IHC staining of Ki67.The growth curve was drawn using tumor volumes against tumor growing days.

### Patients and study design

This study analyzed the mRNA expression of RAP80 in 254 fresh frozen ESCC tissue specimens obtained from two independent cohorts, which include 55 samples from National Cancer Center Hospital in Japan (training cohort) and 199 samples from Nagoya University Graduate School of Medicine in Japan (validation cohort). Patients with histologically confirmed ESCC were eligible. The median follow-up time of ESCC patients in the training cohort was 8.7 years and 3.5 years for the validation cohort. Clinical information was collected from the medical records of each patient and shown in Table S1 and Table S2. According to the rules of the ethics committee, we obtained the written informed consents from all enrolled patients and the study was approved by the institutional review boards of all participating institutions. Patients with radiotherapy or chemotherapy treatment before the surgeries were excluded from this study. Survival time was calculated from the date of surgery to the date of death or last follow-up. The TNM staging was performed according to 7th American Joint Committee on Cancer standards.

### Quantitative reverse transcription polymerase reaction (qRT-PCR)

For the RAP80 expression analysis, High-Capacity cDNA Reverse Transcription Kit (Applied Biosystems, Foster City, CA), and Fast SYBR Green Master Mix (Applied Biosystems, Foster City, CA) were used. The relative expression level of RAP80 was determined by 2^−Δct^ method. GAPDH was used for normalization of RAP80 expression. The primer sequences of RAP80 and GAPDH used are as follows: RAP80 (forward: 5′-GCTTAGTCCTTATGCCAGAGA-3′, reverse: 5′-CCTTGTTACCAACCTCCTTATCT-3′), GAPDH (forward: 5′-CGGAGTCAACGGATTTGGTCGTAT-3′, reverse: 5′-TGCTAAGCAGTTGGTGGTGCAGGA-3′). All assays were performed in duplicate.

### Statistics

Data in the study were represented as mean ± STD from three independent experiments, except that the volumes of tumors from nude mice were represented as mean ± STD of five mice. The Pearson *χ*^2^ tests were applied for the analysis of the expression of RAP80 in the esophageal tissues and its correlation with clinical features. Variables with *p* < 0.05 in the Univariate analysis were used in the subsequent multivariate analysis using the Cox proportional hazards model. Kaplan–Meier survival curves were used to describe the overall survival (OS) distributions of patients with different mRNA levels of RAP80. The log-rank test was used to compare the survival rates between the high and the low groups. The Cox proportional hazards regression was used to obtain univariate and multivariate hazard ratios for the OS. Besides, the two-tailed Students’ *t* test and the ANOVA test were appropriately used according to the statistical guidelines. The significance was indicated as *p < 0.05 or **p < 0.01.

## Electronic supplementary material

Supplementary data
