# Supplementary material for: RAP80 is an independent prognosis biomarker for the outcome of patients with esophageal squamous cell carcinoma
Source: Cell Death Dis. 2018 Feb 2;9(2):146. doi: 10.1038/s41419-017-0177-2 (PMC5833679; doi:10.1038/s41419-017-0177-2)
Supplement: Supplementary file 1 — Supplementary data [file 41419_2017_177_MOESM1_ESM.docx]

**Supplementary data**

**Figure S1.** **A.** EC1 and EC109 transfected with vector or Flag-RAP80 for 48h and then pellets were collected for western blotting assays with indicated antibodies. **B.** Quantitative RT-PCR assays of EC109 and EC1 transfected with shCon. or shRAP80 #1. **C-D.** EC109 (C) and EC1 (D) cells stably infected with shCon. or shRAP80 #1 were transiently transfected with HA or HA-USP13. 48h later, the cell pellets were lysed and subjected to western blotting assays with indicated antibodies. Data from three independent experiments were represented as mean ± STD and statistically analyzed using the ANOVA test. **E.** EC1/shCon. and EC1/shRAP80 #1 cells treated with or without MG-132 were lysed for western blotting assays with indicated antibodies.


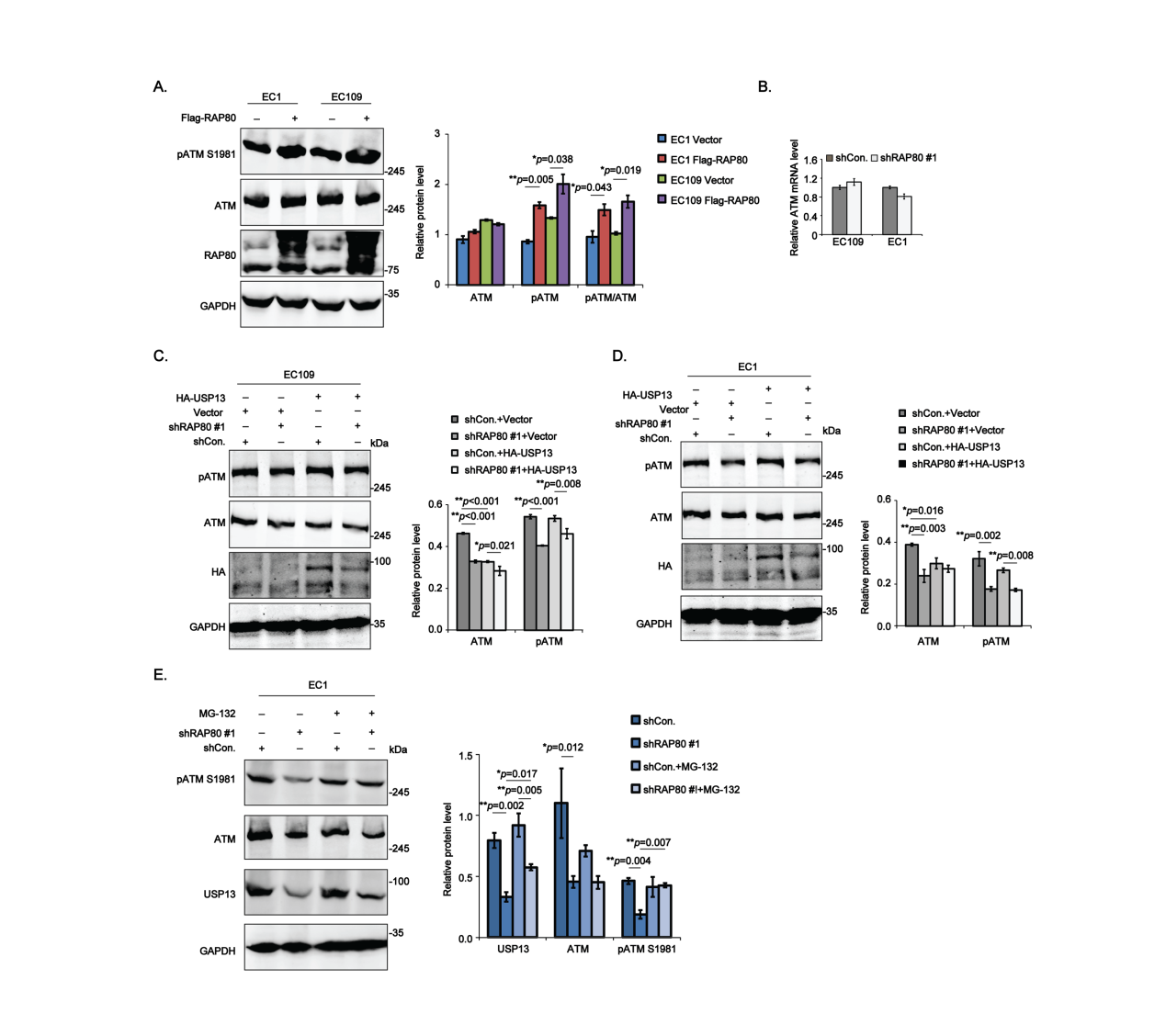


**Figure S2.** The whole protein extracted from EC109 cells treated with or without cisplatin were subjected to immunoprecipitation assays with antibody specific to USP13, followed by the western blotting assays with indicated antibodies.


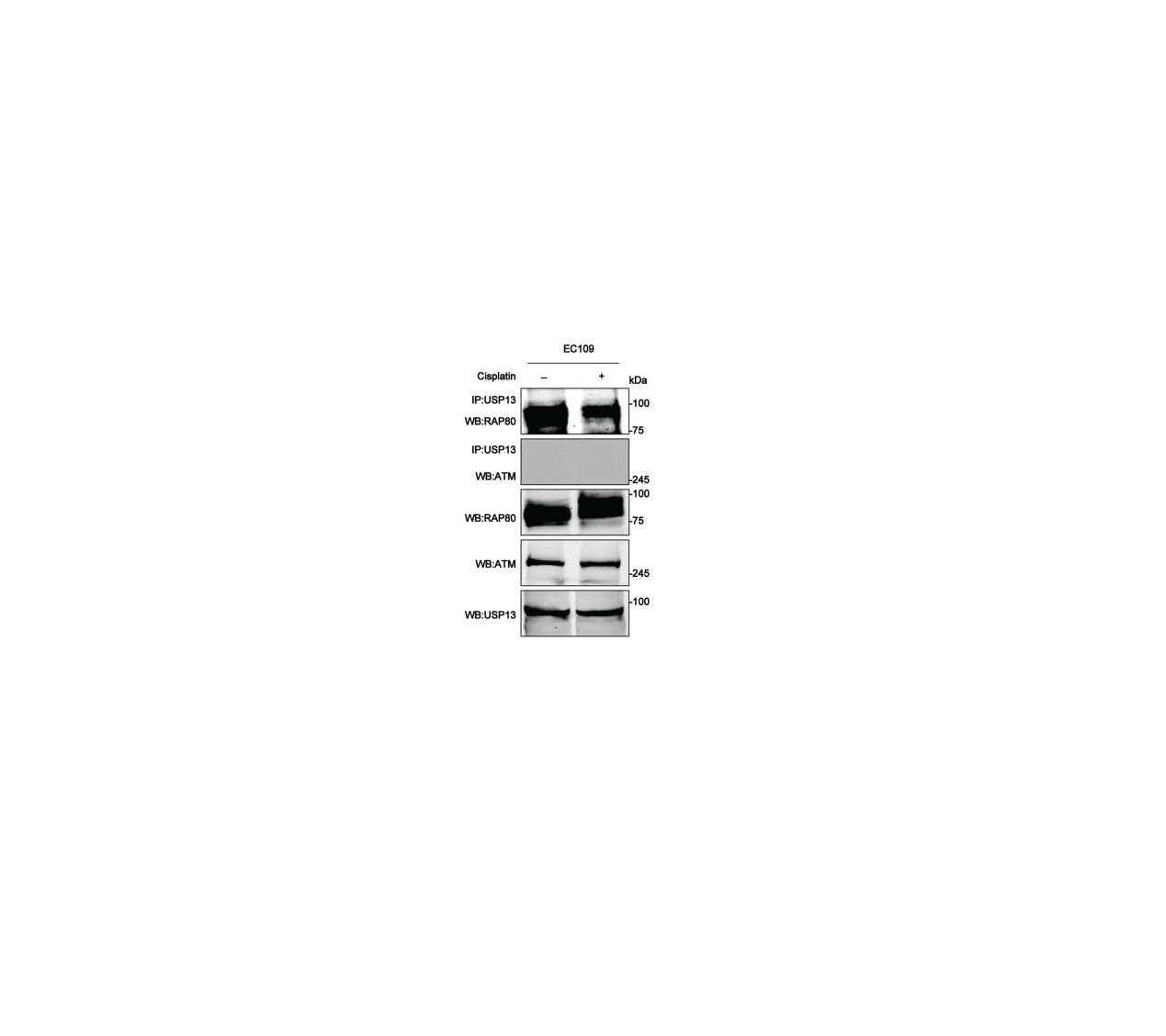


**Table S1. The statistical analysis of relationship between clinical features and the mRNA expression of RAP80 in esophageal tissues in the training cohort.**

| **Characteristics** | **Number** | **expression** | | ***p*-value** |
| --- | --- | --- | --- | --- |
|  | **(n=55)** | **Low** | **High** |  |
| **Gender** |  |  |  | 0.47 |
| **Male** | 47 | 29 | 18 |  |
| **Female** | 8 | 6 | 2 |  |
| **Size (cm)** |  |  |  | 0.178 |
| **<5** | 14 | 11 | 3 |  |
| **≥5** | 41 | 24 | 17 |  |
| **Differentiation** |  |  |  | 0.107 |
| **poor and others** | 18 | 9 | 9 |  |
| **well and moderately** | 36 | 26 | 10 |  |
| **Lymphatic invasion** |  |  |  | 0.799 |
| **absent** | 26 | 17 | 9 |  |
| **present** | 29 | 18 | 11 |  |
| **Nerve invasion** |  |  |  | 0.718 |
| **absent** | 23 | 14 | 9 |  |
| **present** | 32 | 21 | 11 |  |
| **T stage** |  |  |  | 0.792 |
| **T1+T2** | 10 | 6 | 4 |  |
| **T3+T4** | 45 | 29 | 16 |  |
| **LNM** |  |  |  |  |
| **absent** | 12 | 8 | 4 |  |
| **present** | 43 | 27 | 16 |  |
| **TNM stage^b^** |  |  |  | 0.262 |
| **Ⅰ+Ⅱ** | 16 | 12 | 4 |  |
| **Ⅲ+Ⅳ** | 39 | 23 | 16 |  |
| **Distant metastasis** |  |  |  | 0.911 |
| **absent** | 52 | 33 | 19 |  |
| **present** | 3 | 2 | 1 |  |

**Table S2. The statistical analysis of relationship between clinical features and the mRNA expression of RAP80 in esophageal tissues in the validation cohort.**

| **Characteristics** | **Number** | **expression** | | ***p*-value** |
| --- | --- | --- | --- | --- |
|  | **(n=199)** | **Low** | **High** |  |
| **Age** |  |  |  | 0.833 |
| **<60** | 53 | 46 | 7 |  |
| **≥60** | 146 | 125 | 21 |  |
| **Gender** |  |  |  | 0.819 |
| **Male** | 153 | 131 | 22 |  |
| **Female** | 46 | 6 | 40 |  |
| **Size (cm)** |  |  |  | 0.423 |
| **<5** | 141 | 119 | 22 |  |
| **≥5** | 54 | 48 | 6 |  |
| **Differentiation** |  |  |  | 0.209 |
| **poor and others** | 27 | 21 | 6 |  |
| **well and moderately** | 168 | 146 | 22 |  |
| **Lymphatic invasion** |  |  |  | 0.856 |
| **absent** | 52 | 45 | 7 |  |
| **present** | 145 | 124 | 21 |  |
| **N.A.** | 2 |  |  |  |
| **Nerve invasion** |  |  |  | 0.235 |
| **absent** | 122 | 102 | 20 |  |
| **present** | 77 | 69 | 8 |  |
| **T stage** |  |  |  | 0.997 |
| **T1+T2** | 71 | 61 | 10 |  |
| **T3+T4** | 128 | 110 | 18 |  |
| **LNM** |  |  |  | 0.430 |
| **absent** | 70 | 62 | 8 |  |
| **present** | 129 | 109 | 20 |  |
| **TNM stage^b^** |  |  |  | 0.615 |
| **Ⅰ+Ⅱ** | 86 | 75 | 11 |  |
| **Ⅲ+Ⅳ** | 111 | 94 | 17 |  |
| **N.A.** | 2 |  |  |  |
| **Distant metastasis** |  |  |  | 0.241 |
| **absent** | 182 | 158 | 24 |  |
| **present** | 17 | 13 | 4 |  |
